# Cerebrospinal fluid HIV infection and pleocytosis: Relation to systemic infection and antiretroviral treatment

**DOI:** 10.1186/1471-2334-5-98

**Published:** 2005-11-02

**Authors:** Serena S Spudich, Annelie C Nilsson, Nicole D Lollo, Teri J Liegler, Christos J Petropoulos, Steven G Deeks, Ellen E Paxinos, Richard W Price

**Affiliations:** 1 Department of Neurology, University of California San Francisco, USA; 2 Department of Medicine, University of California San Francisco, USA; 3 Gladstone Institute of Virology and Immunology, San Francisco, USA; 4 ViroLogic, Inc., South San Francisco, USA

## Abstract

**Background:**

Central nervous system (CNS) exposure to HIV is a universal facet of systemic infection. Because of its proximity to and shared barriers with the brain, cerebrospinal fluid (CSF) provides a useful window into and model of human CNS HIV infection.

**Methods:**

Prospective study of the relationships of CSF to plasma HIV RNA, and the effects of: 1) progression of systemic infection, 2) CSF white blood cell (WBC) count, 3) antiretroviral therapy (ART), and 4) neurological performance. One hundred HIV-infected subjects were cross-sectionally studied, and 28 were followed longitudinally after initiating or changing ART.

**Results:**

In cross-sectional analysis, HIV RNA levels were lower in CSF than plasma (median difference 1.30 log_10 _copies/mL). CSF HIV viral loads (VLs) correlated strongly with plasma VLs and CSF WBC counts. Higher CSF WBC counts associated with smaller differences between plasma and CSF HIV VL. CSF VL did not correlate with blood CD4 count, but CD4 counts <50 cells/μL associated with a low prevalence of CSF pleocytosis and large differences between plasma and CSF VL. CSF HIV RNA correlated neither with the severity of the AIDS dementia complex (ADC) nor abnormal quantitative neurological performance, although these measures were associated with depression of CD4 counts.

In subjects starting ART, those with lower CD4 counts had slower initial viral decay in CSF than in plasma. In all subjects, including five with persistent plasma viremia and four with new-onset ADC, CSF HIV eventually approached or reached the limit of viral detection and CSF pleocytosis resolved.

**Conclusion:**

CSF HIV infection is common across the spectrum of infection and is directly related to CSF pleocytosis, though whether the latter is a *response to *or a *contributing cause of *CSF infection remains uncertain. Slowing in the rate of CSF response to ART compared to plasma as CD4 counts decline indicates a changing character of CSF infection with systemic immunological progression. Longer-term responses indicate that CSF infection generally responds well to ART, even in the face of systemic virological failure due to drug resistance. We present simple models to explain the differing relationships of CSF to plasma HIV in these settings.

## Background

Frequent abnormalities in cerebrospinal fluid (CSF), including increased white blood cells (WBCs), were recognized early in the AIDS epidemic, not only in patients examined toward the end of their course who suffered neurological complications [1], but also in those systemically and neurologically asymptomatic [2,3]. Indeed, these observations were among the first indicators that the central nervous system (CNS) is an early and common target of systemic HIV infection. While initial studies applying quantitative HIV RNA measurements to the CSF suggested correlation between the CSF HIV RNA (viral load, VL) and the AIDS dementia complex (ADC) [4], subsequent reports have shown that HIV can be found in the CSF throughout the course of infection, beginning with primary infection [5], and that other factors, including the progression of immune dysfunction, are likely important in the development of ADC [6-8]. This has raised the fundamental question of why HIV causes brain dysfunction, manifesting as ADC, only late in the course of infection and only in some individuals [9]. Additionally, because the brain and CSF are separated from the blood by barriers to the transfer of virus, immune defenses and antiviral drugs, there has been considerable concern as to whether local infection in these 'compartments' might be isolated from host defenses and antiviral therapy (ART), leading to both viral persistence and local selection of resistance [10-12]. While CSF and brain infections by HIV are not identical, examination of this easily sampled fluid provides a window into CNS infection [13].

In order to better interpret CSF findings, it is essential to understand what factors contribute to elevated CSF HIV RNA concentrations. How do systemic infection and its progressive damage to the immune system affect the VL in a non-lymphatic compartment like the CSF space? How does CSF infection respond to ART? What is the origin and importance of the CSF cell reaction detected as CSF lymphocytic pleocytosis and how does this cell reaction respond to ART? What is the effect of ART on neurological function in subjects presenting with ADC?

To address these questions and better understand the relationship of the CSF HIV RNA levels to other aspects of infection and clinical findings, we undertook a prospective study of CSF in a broad range of HIV-infected subjects using two complimentary approaches. The first involved cross-sectional analysis of a clinically diverse subject sample. The second longitudinally followed subjects initiating ART. In addition to its direct clinical implications, this longitudinal approach used treatment as an 'experimental' intervention to dissect dynamic aspects of the relationships among these study variables.

## Methods

### Subjects and Protocols

One-hundred subjects were entered into these studies between November, 1996 and June, 2001 in the context of protocols approved by the University of California, San Francisco (UCSF) Committee on Human Research (CHR); follow-up on a few continued until December 2004. Informed consent was obtained from all subjects. In the case of one subjects with ADC, consent was also obtained from his sister with durable power of attorney. Subjects were excluded if they suffered HIV-related or other active CNS diseases except ADC. The cross-sectional analysis targeted a total of 100 subjects, including the baseline observations of subjects starting treatment, along with a previously-reported group who stopped treatment [14,15]. Subjects were clinically stable with the exception of six presenting with a new diagnosis or progression of ADC at the time of study entry.

Treatment decisions were independent of this CSF study. New therapies (either starting *de novo *or representing a change of drugs) were prescribed by subjects' primary care-giver or determined by another clinical trial. Exceptions were five subjects who entered an open-label study of high-dose abacavir (600 mg twice daily) which was completed before abacavir was licensed for clinical use [16,17]. The first subject in this small protocol substituted abacavir for one of the drugs in his existing regimen, while the remaining subjects began this nucleoside reverse transcriptase inhibitor (nRTI) in the context of novel combination ART. Treatment protocols sought to enter subjects with and without ADC, and with plasma VLs ≥ 50,000 HIV RNA copies/mL (cpm), although exceptions were made to this virological entry criterion. Baseline lumbar punctures (LPs) were performed within one week before initiating ART and carried forward as time zero. Most subjects underwent their initial 4–5 LP on-drug studies during the first month, with later assessments at approximately 3 and 6–12 months and then one to three times yearly thereafter.

### LP and CSF analysis

CSF was obtained for study purposes rather than for clinical diagnosis and was processed in standardized fashion as previously described [18,14]. CSF and plasma from each visit were analyzed for HIV RNA concurrently. At the time of the first LP, CSF was also analyzed for neurosyphilis (VDRL) and cryptococcal antigen (all negative). CSF cell counts, differential, protein and albumin levels along with measurement of blood albumin and CD4+ and CD8+ T lymphocyte counts by flow cytometry were performed using routine clinical methods in the San Francisco General Clinical Laboratory.

### Clinical evaluation

Each subject had a baseline clinical evaluation, which included a general and neurological assessment and medical history review; none had current or prior cryptococcal meningitis or any other opportunistic neurological disorder. If indicated by neurological symptoms or signs, subjects had neuroimaging studies to assess for confounding conditions. Subjects underwent a standardized, ADC-focused neurological evaluation leading to ADC diagnosis and staging [19-22]. Diagnosis of ADC conformed to criteria for the AIDS-related cognitive/motor complex outlined by the American Academy of Neurology Task Force [23]. In the presence of any static neurological condition that might interfere with designation of AIDS related cognitive or motor dysfunction (for example, prior head trauma or psychiatric diagnosis), no ADC scale was assigned. Subjects underwent brief quantitative performance testing with a battery of four tasks (timed gait, finger tapping with the dominant hand, grooved pegboard placement with the non-dominant hand, and Digit Symbol test from the WAIS-R) yielding a combined normalized score derived from the mean of individual Z-scores, the quantitative neurological performance Z-score on four tests (QNPZ-4 score), as previously described [24,20,22].

### Virological methods

HIV RNA was measured in cell-free CSF and plasma by the Roche Amplicor Monitor assay (versions 1.0 and 1.5, Roche Diagnostic Systems, Inc., Branchburg, N.J) using the standard and Ultrasensitive extraction methods. The latter has a quantitation limit of 50 and a detection limit of approximately 20 HIV RNA cpm. We used results in the range of 20 – 50 cpm for data reporting and analysis, and assigned a default 'floor' value of 19 (log_10 _1.28) cpm for values below the detection limit. Concurrent paired CSF and plasma samples were treated identically and run at the same time. HIV RNA concentrations were transformed to log_10 _values for all analysis. Because limited serial sampling during the acute phase of treatment-induced viral decay precluded complex modeling, estimates of acute-phase HIV-RNA half-lives were derived by assuming simple exponential decay during an initial phase (days 0 – 11) as previously described [25]. While the acute phase of plasma viral decay is shorter than 11 days, we used this extended period for rough comparison because of the limited sampling in some subjects. The acute decay rate, λ, for each subject was estimated by least-square regression of measurement time (including baseline) on a subject's log_10 _HIV-RNA values. The subject's acute-phase HIV-RNA half-life was log_10_2/λ.

### Antiretroviral drug resistance

Antiretroviral drug resistance was assessed by both phenotypic and genotypic methods in selected subjects using the PhenoSense™ HIV assay (ViroLogic Inc., South San Francisco, CA) to analyze functional susceptibility in a recombinant assay and mutations in the reverse transcriptase (RT) and protease (PR) regions [26]. Phenotypic susceptibility results were reported as fold-change in the 50% inhibition concentration (IC_50_) relative to a wild-type virus reference standard, while genotypic analysis compared RT and PR sequences in the blood or CSF HIV populations to the reference wild-type (e.g., NL4-3).

### Statistical analysis

Because of the skewed distributions of several study variables, unless otherwise indicated, the median and interquartile range (IQR; 25th percentile – 75th percentile) were used for descriptive statistics, and nonparametric tests were used for comparisons. In the cross-sectional analysis, all *p*-values were two-sided, with *p*-values < 0.01 considered significant. Statistics were performed using SPSS 11.5 (SPSS Inc., Chicago, IL), or Prism 4.0 (GraphPad Software Inc, San Diego, CA).

## Results

### Study subject demographics

Of the 100 subjects in the cross-sectional study, 65 were recruited into a *cross-sectional only *group and studied only once, 26 were in a *longitudinal treatment *group and were followed with serial LPs, and nine subjects were in a *longitudinal Structured Treatment Interruption (STI) *group and are reported elsewhere [14,15]. However, as two of these nine STI group subjects were studied after they restarted treatment, they were also included among the longitudinal treatment group, bringing the total number of patients in the longitudinal treatment group to 28. Table 1 provides a summary of the salient clinical and laboratory variables in the 100 subjects and also divides them according to ART treatment status. Additionally, Table 1 summarizes data from a subgroup of patients on ART who were considered *treatment failures*, as defined by plasma HIV RNA concentration >500 RNA cpm.

**Table 1:** Baseline Characteristics of Study Subjects, including subgroups.

	**Sex:**								**HIV-1 RNA**			
										
	**N**	**Age**	**M:F**	**CD4+**	**Duration of Infection**^**a**^	**CDC Stage C3**^**b**^	**ADC Stage**	**QNPZ-4 Score**	**Plasma**	**CSF**	**P-C log_10 _Diff.**	**CSF WBC**
	
		(yrs)	(ratio)	(cells/mm^3^)	(mean yrs +/- SD)	(percent)			(log_10_RNA copies/mL)			(cells/mm^3^)
**Total**	100	39.0 (36.0–45.0)	91:9	181.5 (48.8 – 285.3)	9.7 (+/- 5.60)	70.3%	0 (0 – 1)	-0.50 (-1.53 – 0.21)	4.73 (3.52 – 5.15)	2.74 (1.48 – 4.00)	1.30 (0.19 – 2.32)	1.0 (0.0 – 4.0)
*ADC ≥ 1 = 30.0%*												
**Subdivision by Treatment Group and Effect**												
***Off Treatment***												
	46	38.0 (33.5 – 43)	42:4	195.0 (33.5 – 307.5)	7.5 (+/- 5.80)	65.2%	0 (0 – 0.5)	-0.46 (-1.35 – 0.26)	4.93 (4.53 – 5.49)	3.61 (2.57 – 4.40)	1.19 (0.42 – 21.7)	2.0 (0 – 11.3)
*ADC ≥ 1 = 23.1%*												
***On Treatment (Total)***												
	54	43.0 (38.0-29.3)	49:5	181.5 (76.0 – 275.5)	11.09 (+/- 5.08)	75.9%	0.5 (0 – 1.0)	-0.51 (-2.62 – 0.20)	3.40 (1.85 – 4.80)	1.66 (1.28 – 3.07)	1.37 (0.0 – 2.42)	1.0 (0.0 – 2.0)
*ADC ≥ 1 = 36.6%*												
***On Treatment Failures***												
	36	40.0 (38.0 – 47.8)	32:4	166.5 (48.3 – 276.5)	11.24 (+/- 4.18)	75.0%	0.5 (0 – 1.0)	-0.42 (-2.40 – 0.25)	4.73 (3.99 – 5.04)	2.45 (1.45 – 3.64)	2.19 (1.29 – 2.59)	0.5 (0.0 – 2.8)
*ADC ≥ 1 = 37.0%*												

Values are medians with IQR in parentheses beneath, unless noted. P-Clog_10 _Diff. is the difference in plasma and CSF log_10 _HIV concentrations.

^a^Data available for 49 of the 100 subjects.

^b^Data available for 81 of the 100 subjects; categorization based on the 1993 Centers for Disease Control HIV classification system.

Reflecting the demography of the local epidemic, over 90 percent of our subjects were men, with a median age of 39 years. The majority of the cross-sectional only sample was on ART, though VL was undetectable in plasma in only eight of these 65 subjects, reflecting a bias toward entry of subjects with virological failure.

While most of the 28 subjects in the longitudinal treatment group were ART-naive or had limited prior therapy, seven were on therapy, changing or adding one or more drugs at baseline. Twenty-four of 80 subjects without confounding neurological conditions (30%) were diagnosed with ADC: 13 subjects with ADC Stage 1, 8 subjects with ADC Stage 2, and 3 subjects with ADC Stage 3. Most had stable neurological impairment and clinically 'inactive' CNS disease (exceptions are discussed below). The prevalence of ADC was the principal reason for the overall median QNPZ-4 score below "normal".

### Cross-sectional analysis

#### CSF HIV RNA

**Figure 1:**
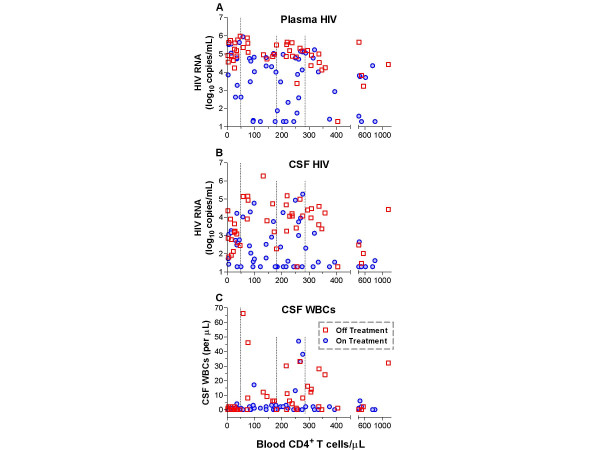
Baseline distributions of HIV concentrations and CSF WBCs in relation to blood CD4 cell counts in the 100 subjects analyzed cross-sectionally. Individual panels show: (A) plasma HIV RNA, (B) CSF HIV RNA, and (C) CSF WBC counts in relation to blood CD4 counts. The vertical dotted lines in each panel separate the subjects into blood CD4 quartiles. The Symbol key appears in the bottom right panel, red boxes indicate patients off treatment, while blue circles indicate those on treatment.

In the cross-sectional evaluation, HIV RNA was characteristically lower in CSF than in plasma, and the VL differences between the two compartments, which we express here and below as the *ΔPlasma:CSF *(log_10 _plasma HIV RNA – log_10 _CSF HIV RNA), varied widely, ranging from -1.32 to 4.08 log_10 _cpm (Table 1). CSF VL was higher than plasma VL in only 11 of 100 subjects, with a difference of >0.50 log_10 _cpm found in only 3 of these 11 subjects. Figure 1 shows the distribution of the plasma (A) and CSF (B) HIV RNA in relation to the blood CD4 cell counts in the 100 subjects. Division of the results into CD4 quartiles shows that the cohort did not distribute evenly among CD4 values, so that one quarter of the subjects had CD4 counts below 49 cells/μL.

The treated subjects had lower VLs in both compartments (Table 1, Figure 1; p < 0.001 for both; Kruskal-Wallis test). To assess the effects of ART on CSF HIV in the setting of treatment failure, we analyzed the results in the subjects on ART who had plasma HIV VLs above 500 cpm and include this subgroup in Table 1. The median plasma VL in this subgroup was similar to that of the off treatment group, though these groups differed statistically (*p *= 0.017, Mann-Whitney). More notably, the median CSF values differed by more than 10-fold (p = 0.001), and therefore the median ΔPlasma:CSF of the failure group was greater than the median for subjects off treatment. This *post hoc *analysis raises the question of whether 'failed' treatment might alter the relationship of CSF to plasma HIV, and indicates that despite plasma HIV escape, treatment still had an effect on CSF.

Overall, CSF HIV RNA correlated with only two other variables examined, the plasma VL and the CSF WBC count (*p *< 0.001 for both, Spearman's rho 0.629 and 0.516, respectively). In contrast, CSF HIV did not correlate with the blood CD4+ cells, ADC stage or QNPZ-4 scores. However, as shown on Figure 1, plasma VLs were highest in subjects in the first CD4 quartile (<49 cells/μL). The plasma VL in this quartile was 5.03 log_10 _cpm with IQR 4.67 – 5.57, while the median plasma VL of the remaining 75 subjects was 4.35 log_10 _cpm with IQR 2.92 to 4.98 (*p *= 0.001). In contrast, the CSF VLs of subjects in this first quartile (median 2.75 log_10 _cpm, IQR 2.15 – 3.20) did not differ from those of the remaining 75 subjects (median 2.64 log_10 _cpm, IQR 1.28 – 4.19) (*p *= 0.650). The combined effect of higher plasma VL and similar CSF VL in the first quartile compared to the other subjects resulted in a greater median ΔPlasma:CSF for subjects in the first quartile (2.25 log_10 _cpm, with IQR 1.75 – 2.95), relative to the median for the remaining 75 subjects (0.800 log_10 _cpm, IQR of 0.00 – 1.97) (*p *= 0.001).

#### CSF WBC counts

Twenty-four subjects had abnormal CSF WBC counts (>5 cells/μL), composed of 85–100 percent lymphocytes, with the remainder mononuclear cells. All 24 were asymptomatic, despite a median count of 15 cells/μL (IQR 9 – 32 cells/μL, range 6 – 66 cells/μL). The CSF WBC count did not significantly correlate with plasma VL.

**Figure 2:**
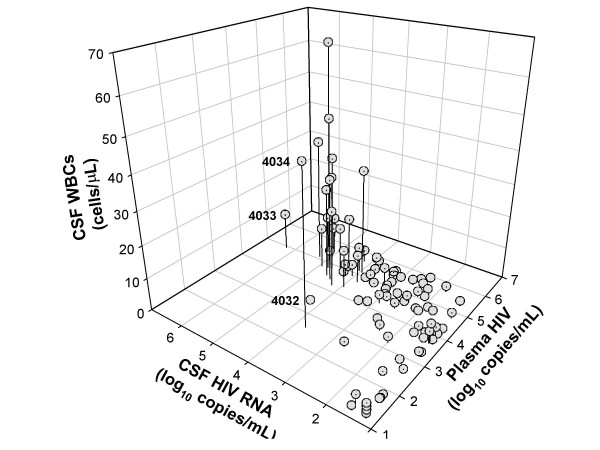
Three-dimensional plot showing relationships among plasma and CSF HIV RNA concentrations and CSF WBC counts. Subjects with CSF pleocytosis had highest CSF VLs. In turn, higher CSF VLs were noted chiefly in those with elevated plasma HIV (>10,000 cpm). The numbers near three of the data points identify the three subjects with substantially higher CSF than plasma HIV RNA (see text).

Figure 2 shows the relationship between CSF WBC count and plasma and CSF HIV VLs using a three-dimensional plot. The highest CSF VLs were in subjects with *both *pleocytosis and high plasma VLs (generally ≥ 4.0 log_10 _cpm). These were also the subjects with the highest CSF VLs. By contrast, many subjects with similarly high plasma VLs *without *pleocytosis had lower CSF VLs. Three subjects with CSF VLs that substantially exceeded those of plasma are indicated by subject number in Figure 2 and are discussed below. Subjects with CSF WBC counts ≥ 10 cells/μL had a median VL difference between the two fluids of 0.150 log_10 _cpm (IQR -0.115 – 0.425 copies) – far below the difference seen in the group overall.

CSF WBC counts showed only a modest correlation with CD4 counts (*p *= 0.01; rho 0.254), Figure 1C shows that this correlation related chiefly to differences between the subjects in the lowest CD4 quartile (less than 50 cells/μL) and the remaining subjects with higher CD4 counts. The median WBC count for first quartile was 0 cells/μL (IQR 0 – 1.0) while that for the remaining subjects was 2.0 cells (IQR 0 – 8.3 cells) (*p *= 0.009; t-test).

#### Neurological status

Neither the ADC stage nor the QNPZ-4 score correlated with the CSF or plasma VLs across the cross-sectional group, though these two measures correlated strongly with each other (*p *< 0.001, rho -0.746), and both also correlated with the CD4 count (*p *< 0.001 for both, rho = -0.515 for ADC stage and 0.467 for QNPZ-4). For all ADC subjects, the CD4 median was 67.5 cells/μL (IQR 28.0 – 124.5); for the untreated ADC subgroup, the median was 35 (IQR 14.5 – 67.5) cells/μL, and for those on ART, the median was 95 cells/μL (IQR 36 – 205).

Only 6 subjects were judged to have active ADC. Two of these were treatment failures with active disease despite ART, while the other four were off ART at presentation. Among the six were the three subjects designated as outliers in Figure 2 because of VLs higher in CSF than in plasma: subject 4034 with ADC Stage 1, and subjects 4032 and 4033 with Stage 2. While two had elevated CSF WBC counts consistent with the general positive correlation between of high CSF VL and CSF WBC count, subject 4032 did not. His CSF VL was high (19,700 cpm), his plasma HIV level was nearly tenfold lower (2,800 cpm), and the usual Δplasma:CSF was reversed despite acellular CSF. While he was prescribed nelfinavir, abacavir and zidovudine, it was suspected that he was not consistently taking full dosage, and genotypic resistance studies showed no evidence of significant resistance-associated mutations in the HIV PR or RT regions (not shown).

### Longitudinal treatment studies

Longitudinal analysis involved 28 subjects who were followed with repeated LPs after initiating or modifying treatment. This is an extension of our earlier published study, adding 13 subjects and prolonging the period of follow-up for several of the 15 subjects previously described [25]. Twenty of these subjects were either treatment-naïve, had limited ART exposure, or had been fully suppressed in the past before stopping therapy and were therefore anticipated to respond well to ART. The remaining eight subjects had failed their previous treatment and were either changing therapies or restarting ART after a hiatus, with addition or substitution of one of more drugs at entry into the study.

**Figure 3:**
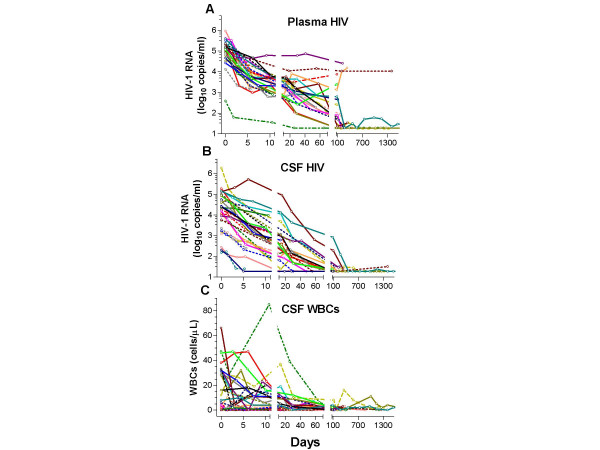
Responses to ART. The panels show the individual subject plots of changes in the plasma (A) and CSF (B) HIV RNA concentrations and (C) WBC counts after treatment with the time axis broken into three segments showing initial, intermediate and longer-term outcomes.

Subjects underwent multiple LPs (median 6, IQR 5–8). Figure 3 shows the course of their HIV RNA levels in plasma (A) and CSF (B) along with changes in CSF WBCs (C) during the follow-up. At baseline, the plasma VLs were both higher and within a narrower range than those of CSF.

#### Early-phase CSF virological responses

The graphs in Figure 3 are divided into 3 temporal segments. Visual comparison of the initial segment (days 0–11) suggests that early viral decay was slower in CSF than in plasma for some subjects. Using previously described methods that apply linear regression to the log_10 _HIV RNA values in CSF and plasma [25], and restricting comparison to subjects who began multidrug regimens and exhibited rapid initial plasma decay, we derived CSF:plasma decay ratios in 18 of the subjects who had at least 3 LPs between days 0 and 11.

**Figure 4:**
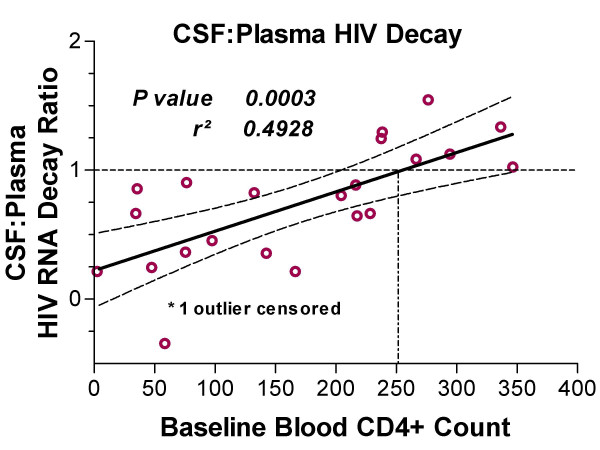
Relation of CSF:plasma early-phase decay ratio to baseline blood CD4 cell counts. The regression line and 95% confidence intervals were plotted after censoring one subject with CD4 = 1,140 cells/μL. The p-value and *r*^*2 *^of this regression analysis are shown on the figure, while the results of nonparametric analysis are discussed in the text. The horizontal broken line designates the point at which plasma and CSF decay are equal (ratio of 1) and the vertical broken line signals the point where this crosses the regression line – near a blood CD4 count of 250 cells/μL.

The CSF:plasma HIV decay ratios were variable (median 0.82, IQR 0.41–1.10). Exploration of the relationship of these ratios to baseline variables showed a high correlation with only the blood CD4 T lymphocyte count (*p *< 0.000, rho -0.744), and that neither the baseline VLs themselves, the CSF WBCs, nor the ADC stage had a significant effect. Figure 4 shows the relationship between the CSF:plasma viral decay ratio and the baseline CD4 counts and includes a regression line that shows equal decay in plasma and CSF (a CSF:plasma decay ratio value of 1) near CD4 = 250 cells/μL, with slower decay at lower CD4 counts. This decay difference related to CD4 count suggests a change in the character of CSF infection with more advanced systemic infection.

#### Longer-term virological and WBC responses in CSF

Despite slower initial decay in CSF compared to plasma in some subjects, the longer-term effects of ART on CSF HIV RNA in this group were excellent, and all subjects reached or approached the limit of detection over the period of observation (Figure 3A and 3B). As shown in Figure 3C, treatment also eliminated the CSF pleocytosis in all subjects with elevated baseline WBC counts.

**Figure 5:**
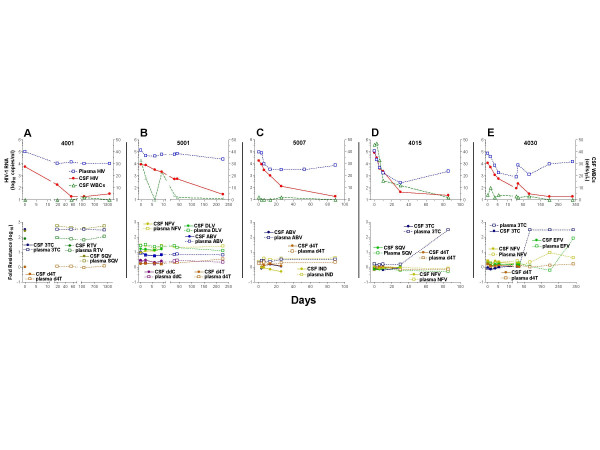
Dissociated CSF and plasma HIV RNA responses in five subjects. Each of these subjects achieved near or full CSF viral suppression despite an incomplete plasma response. The top panels of each pair show CSF and blood HIV RNA and CSF WBC values (A). The lower panels graphically depict the phenotypic resistance profiles as fold change in susceptibility to the drugs these subjects were taking during the study compared to reference wild type on a log_10 _scale [26]. See the text for discussion.

Particularly notable in the treatment group were five subjects who achieved CSF HIV suppression despite persistent plasma viremia. Their CD4 counts were similar to the larger group (median 205 cells/μl, range 77 – 269). Four were neurologically normal, while the fifth (subject 5007) had a diagnosis of ADC based on longstanding and clinically static myelopathy. The plasma and CSF HIV and WBC changes for these five subjects with *dissociated longer-term responses *are shown in Figure 5 (upper panels), along with results of phenotypic resistance testing for the drugs that they were taking (lower panels). The antiretroviral medication histories and genotypic resistance mutations detected in the two fluids of these five subjects are presented in Table 2 [see Additional file 1]. All were treatment-experienced when entering the study; three (4001, 5001 and 5007) were on therapy and changing or modifying their regimens, while the remaining two (4015 and 4030) were off ART at the study start and were initiating new regimens.

The results of genotypic and phenotypic drug resistance analysis of plasma and CSF indicate that persistent plasma viremia was associated with drug resistance that was either demonstrable at baseline (subjects 4001, 5001 and 5007) or emerging during the period of observation (4015 and 4030). In two subjects (5001 and 5007) with baseline resistance, minor differences in genotypic mutations suggested at least partial compartmentalization, with CSF viruses more drug-susceptible than the predominant plasma quasispecies. Phenotypic drug susceptibility testing in Subject 5001 (Figure 5B) showed resistance to drugs in his regimen in both compartments, including resistance to abacavir. Minor differences in resistance mutations in the two fluids (Table 2 [see Additional file 1]) were likely insufficient to explain his greater CSF response. Genotypic analysis in Subject 5007 (Table 2 [see Additional file 1]) suggests a mixed population of drug susceptible and resistant viruses in CSF samples (for example M41M/L, L74L/V and T215T/N/S/Y) compared to a predominantly resistant population in plasma (for example M41L, L74V and T215Y). Perhaps greater susceptibility of a 'compartmentalized' CSF virus population contributed to the greater CSF response (Table 2 [see Additional file 1] and Figure 5C). Interestingly, the highly resistant plasma HIV population did not 'overflow' into the CSF and completely alter its resistance profile.

The other three (4001, 4015, and 4030) showed no evidence of compartmentalization at baseline, with similar susceptibility in both plasma and CSF virus populations. In Subject 4001 (Figure 5A), high-level resistance to lamivudine, saquinavir and ritonavir raises the question of whether the therapeutic effect on CSF may have related principally to stavudine, though this drug was insufficient to suppress plasma HIV. The initial samples of both plasma and CSF from Subject 4015 (Figure 5D) showed mixed populations of lamivudine resistance (M184M/V), from which emerged the resistant quasispecies (M184V) at the time of viral rebound (Table 2 [see Additional file 1]). In Subject 4030 (Figure 5E) phenotypic and genotypic resistance testing showed nearly identical susceptibility in CSF and plasma, and wild-type genotypes in both compartments. At day 85 and afterwards, his plasma HIV showed increasing resistance to lamivudine, nelfinavir and efavirenz (resistance to nevirapine increased similarly, not shown), with susceptibility only to stavudine, though genotyping showed the emergence of a stavudine mutation, T215Y. In addition to the disproportionate CSF HIV response, CSF WBC counts were also suppressed in these subjects. This was most remarkable in subjects 4015, with more than 45 cells/μL, and 5001, with 33 cells/μL at baseline; in both, the pleocytosis resolved despite sustained plasma HIV.

#### Neurological responses to ART

**Figure 6:**
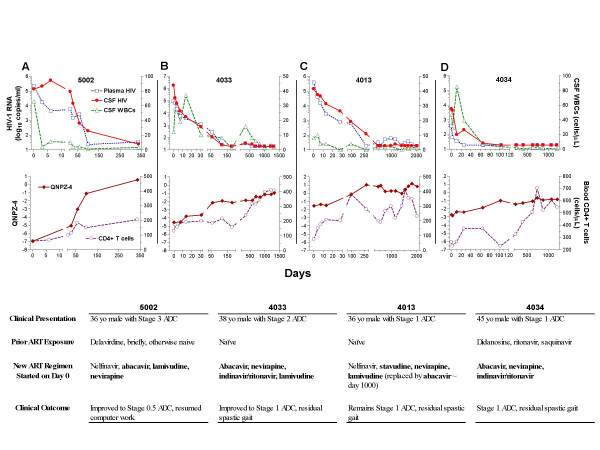
Longitudinal follow-up of four subjects presenting with new-onset or progressing ADC. The upper panel of each pair shows the plasma and CSF VL responses along with CSF WBC changes, and the lower panels show treatment effects on the QNPZ-4 scores and the blood CD4 counts. The table below each graph indicates clinical features of each respective subject. Antiretroviral medications considered able to penetrate the CSF [10] are indicated in bold font. The key to the symbols for all graphs are shown to the left of panel set (A).

In the 24 treatment-group subjects without active ADC at baseline, QNPZ-4 scores were stable or showed small increases over the course of observation (not shown). The four subjects in the treatment group who presented with active ADC (5002, 4033, 4013, and 4034) showed both clinical improvement and distinct increases in this performance score in response to initiation or change of antiretroviral therapy. The clinical histories, antiretroviral therapy regimens, laboratory measurements, and QNPZ-4 scores of these subjects are presented in Figure 6. Of note, genotypic resistance testing, available at baseline for Subject 4034 (Figure 6D) who developed ADC on treatment with an unusual regimen, showed concordance in CSF and plasma with no resistance mutations in the RT and only L63P, A71A/V and V77I changes in the PR. These findings are consistent with insufficient drug potency and poor drug penetration into the CSF, and might suggest that the CNS served as a major site of viral replication in this subject. In response to a change to abacavir, nevirapine and indinavir/ritonavir, he achieved virological suppression in both and an improvement in the speed and clarity of his cognition.

## Discussion

The CSF found in the ventriculo-leptomeningeal space is separated from systemic sources of virus, immune defenses, and antiviral drugs, and is easily sampled by lumbar puncture. Thus, study of CSF HIV infection can serve a valuable role in understanding the dynamics and mechanisms of infection within an isolated tissue compartment. Further, CSF infection serves as both a model of and window into brain infection, providing important insight into viral neuropathogenesis. This CSF 'compartment' can be viewed as parallel to the brain compartment, sharing some of its barrier features. However, exchanges between CSF and blood may differ from those between brain and blood, and as a result, tissue responses may differ from CSF responses in important ways [27]. More directly, the CSF may reveal brain processes as a consequence of its intimate contiguity and function as an extracellular 'sink' where molecules produced in the brain (or along its perivascular spaces) that have diffused into the CSF can be sampled [8].

The studies described here used complimentary cross-sectional and longitudinal approaches to show that CSF infection is a nearly universal facet of the ecology of systemic HIV infection. CSF HIV RNA concentrations correlated with those of plasma, but were characteristically lower. The median difference between plasma and CSF HIV VLs (Δplasma:CSF) was 1.30 log_10 _cpm though this difference also varied considerably, ranging from -1.32 to 4.08 log_10 _cpm. Our results extend previous reports [4,6,7,28-31] and focus on the factors that modify CSF infection in relation to plasma VL. CSF infection is importantly influenced by the CSF WBC response, the degree of systemic immunological progression as measured by blood CD4+ T cells, antiretroviral treatment and drug resistance, and the presence of active ADC.

### CSF WBCs

CSF WBC counts were commonly elevated in this series, confirming findings reported earlier in the epidemic (e.g., [2]). Our steady-state cross-sectional and treatment-related longitudinal observations confirm that this CSF pleocytosis is directly linked to HIV infection itself, rather than to another cause such as undiagnosed opportunistic infection. Specifically, the cross-sectional analysis showed that CSF WBC counts were highly correlated with CSF HIV RNA concentrations (Figure 2) though not with the plasma VL. A similar association of CSF HIV with CSF WBC count has previously been reported by several groups [32,8,33]. The longitudinal studies of treatment indicated this association even more clearly, revealing resolution of baseline pleocytosis in all those beginning ART (Figure 3C). This association is also supported by our previous report that about half of subjects undergoing STI develop a brisk CSF pleocytosis upon stopping therapy [14].

While this combined experience indicates that CSF VLs and WBC counts are related, it begs the fundamental mechanistic question of whether the CSF WBCs actually *contribute to *raising the CSF VL, or alternatively, represent only a *response to *high CSF virus. We discuss these two alternative hypotheses in relation to models of CSF infection below.

### Change in the character of CSF infection with disease progression

Two of the observations reported here show a change in the relation of CSF infection to systemic infection as the latter progresses (as indicated by reduction in blood CD4 lymphocyte counts). First, in the cross-sectional study the Δplasma:CSF values in subjects with CD4 counts below 50 cells/μL (the first CD4 quartile of the group) were significantly larger than those of the remaining subjects with more preserved CD4 counts. This was related to higher plasma VLs, but without concomitant increase in CSF VLs; rather CSF VLs for these subjects were similar to those of the remaining subjects. This quartile also had lower CSF WBC counts. This observation confirms the impression of earlier investigators that CSF cell counts decrease in those with more advanced systemic infection [2]. We discuss later how these two observations, higher Δplasma:CSF and reduced CSF WBC counts, might be linked.

The second difference was in the rates of viral decay during the initial phase of therapy in CSF compared to plasma. Viral decay in CSF was slower than in plasma in subjects with lower CD4 counts, while decay rates in the two fluids were more often equal at higher CD4 counts. This finding extends our earlier report of this association [25]. It agrees, in part, with the findings of Ellis and colleagues [34] of a similar CD4 effect in a smaller series. However, this association of early decay differences with CD4 counts is at variance with a report by Eggers and colleagues [35] who correlated slower CSF decay after therapy with the presence of ADC or HIV encephalitis. Indeed, our results suggest that slower CSF decay was not confined to ADC subjects, but also occurred in neurologically asymptomatic subjects with low CD4 counts as noted by Ellis et al [34]. Further elucidation of these associations and the reasons for the minor differences in the experiences of different research groups will require a larger, more varied population sample, or combined analyses of the experience from several centers. Whatever the precise association, these observations suggest that the character of CSF infection changes with disease progression. In those with less advanced systemic disease, CSF infection often responds to potent ART as rapidly as blood infection. In those with more advanced systemic disease, with or without ADC, CSF HIV responds more slowly to antiviral therapy.

### Overall effects of ART on CSF HIV infection

Before undertaking this study, we were concerned that CSF infection might not respond as well as systemic infection to treatment because of restricted penetration of many antiretroviral drugs [10]. This was not borne out by our observation that combination ART had a favorable impact on CSF HIV infection in the population of treated subjects. In the cross-sectional sample, both the plasma and CSF VLs were higher in untreated (median 4.93 and 3.61 log_10 _cpm in plasma and CSF, respectively) than in treated (median 4.00 and 1.66 log_10 _cpm in plasma and CSF) subjects (*p *< 0.001 for both variables compared by t-test). As noted also, HIV RNA was below the detection limit in a greater proportion of the CSF (38.9%) than plasma samples (18.5%). Given that the CSF HIV responded to therapy at least as effectively as plasma HIV, a relatively impaired CSF penetration of antiretroviral drugs seems unlikely to have influenced our findings. However, we did compare the CSF penetration of the antiretroviral regimens between those 'failing' to achieve virological suppression in plasma, and those with viral suppression, using a summed score in which each drug known to penetrate CSF counted as one and those not penetrating well counted as zero (data not shown). The means of the summed scores in the two groups (1.86 +/- 0.93, and 2.33 +/- 1.19, respectively) were not significantly different (*p *= 0.115). Of note, high CSF HIV was noted in rare treated subjects, sometimes exceeding plasma levels, as in subject 4034 who was treated with an unusual, poorly CSF-penetrating drug regimen [36] but responded well to an altered regimen. A second example of higher CSF than plasma HIV was subject 4032 who was suspected to be poorly adherent to his medications; genotypic testing supported this hypothesis by showing that his treatment failure was not due to drug resistance. Thus, although classified as *on therapy*, he likely was on intermittent and ineffective ART at best [37]. Overall, our experience suggests that effective CSF viral suppression is the general rule when plasma virus is suppressed.

The effectiveness of ART was clearly evident in the longitudinal treatment series. While the initial rate of CSF HIV decay lagged behind that of plasma in some subjects, all those with long-term follow-up achieved gratifying HIV reduction in this compartment with a time course generally proportional to their baseline level (Figure 3). None of the 28 patients showed persistent CSF virus in the face of undetectable plasma VL. The responses in subjects without previous treatment were similar to those reported by Polis and colleagues [30]with a protracted course but excellent suppression at 6 months. Our results also correlate well with the longer-term outcomes in more heterogeneous cohorts reported by others [38,39,35].

### Effect of treatment of CSF infection in the presence of drug resistance

In addition to the generally favorable effects of ART on CSF infection, we observed an intriguing, salutary facet of treatment response. Namely, CSF infection showed a proportionally greater response to treatment than plasma infection in the face of antiviral drug resistance. Since drug resistance is the principal reason for long-term treatment failure, this may have very important implications for the prevention and treatment of CNS HIV infection and ADC. This superior effect of ART on CSF infection was noted in both the cross-sectional study and in the longitudinal treatment study. Moreover, it had been anticipated in our previous observations of CSF rebound in subjects with virological failure who underwent STI [18].

To examine this issue in the cross-sectional group, we compared the 36 subjects who were on therapy but had plasma VLs above 500 cpm (defined as *treatment failures*) with the 46 *untreated *subjects. While these two groups had similar plasma VLs (median 4.73 log_10 _cpm, IQR 3.99 – 5.04 in the treatment failure group, and 4.93 log_10 _cpm, IQR 4.53 – 5.48 in the untreated; p = 0.072 by t-test), their CSF HIV RNA levels differed substantially (2.45 log_10 _cpm median, IQR 1.45 – 3.64 log_10 _cpm in the treatment failure group, and 3.61 median, IQR 2.57 – 4.40 in the untreated; p = 0.002). As a result, the Δplasma:CSF was about ten-fold higher in the treatment failure (median, 2.19 log_10 _cpm, IQR 1.90 – 2.58) than in the untreated group (median 1.18 log_10 _cpm, IQR 0.42 – 2.16; p = 0.025); this 100-fold difference between plasma and CSF was similar to that reported by Stingele and colleagues in a study of paired specimens evaluating resistance mutations [29]. Thus, *'treatment failure' *in the cross-sectional group was often associated with proportionally greater VL reductions in CSF than plasma.

This effect was further illustrated in the 'dissociated' CSF responses of the five treatment subjects who failed to clear virus in the plasma but responded completely or nearly completely to treatment in the CSF (Figure 5, Table 2 [see Additional file 1]). Their CSF HIV responses were both proportionally greater and more prolonged than those of plasma. Analysis of drug susceptibility in these 5 subjects showed that the main reason for continued plasma viremia was drug resistance. At least two patterns of responses were noted: 1) resistance at baseline leading to an initial small reduction in viremia and a new plateau (subjects 4001, 5001 and 5007, who switched medication at the start); and 2) a greater initial response followed by later partial recrudescence (subjects 4015 and 4030 who had been off therapy for some time after treatment failure before starting back on a new regimen). In the first two subjects, resistance was well established at baseline, while in the second two it emerged during the study, presumably from archived resistant strains [40]. Subject 5007 (Figure 5C) may have had a greater degree of compartmentalization of resistant virus. Genotypic testing revealed a plasma viral population with resistance-associated mutations at several amino acid sites (e.g., Protease mutation L90M and Reverse Transcriptase mutations M41L, K103N, Y181C, and T215Y). Testing of the CSF viral population revealed mixtures of wild-type with resistance-associated amino acids at all these sites. Such compartmentalized resistance with differences in drug susceptibility between CSF and plasma clearly occurs [41,29,42]. Notably, however, in the context of such systemic and CSF resistance, ART can be proportionally more effective in suppressing CSF infection than in treating systemic infection.

In fact, this greater effect on CSF than plasma in the face of drug resistance and treatment failure was predicted by observations on subjects failing ART who undertook STI [14,18]. In some of these subjects, the baseline CSF VLs were 10- to 1000-fold lower than baseline plasma VLs, but upon STI, rose to levels near or equal to those of plasma, indicating that the 'failed' therapy had suppressed the HIV to a greater extent in CSF than in plasma.

### Models of blood-CSF virus and cell exchange and compartmentalization

**Figure 7:**
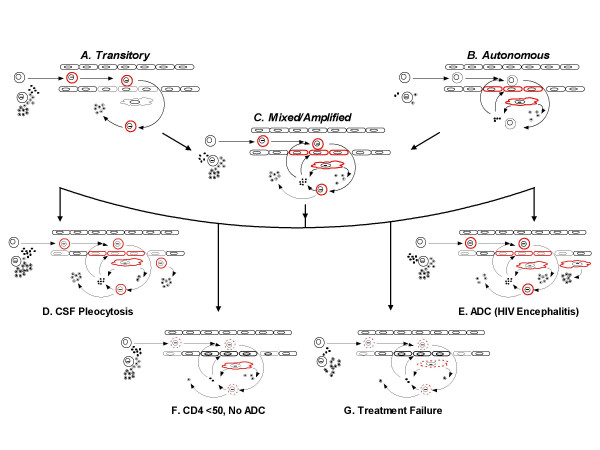
Model of CSF HIV infection. The diagram provides a simple schematic of hematogenous infection with T cells (including HIV-infected CD4 cells) and intrathecal macrophages separated by the blood-brain endothelial barrier. The model presumes that virus reaches the CNS principally within infected cells. T cells are shown as round cells, either infected (bar within nucleus) or uninfected (no bar). Similarly, the macrophages are shown as flat, elongated cells with or without infection (again, bar in nucleus). Both virus (circles with central dot) and cytokine/chemokine (smaller solid circles) are produced or provoked by infection on both sides of the barrier. Cells particularly involved in the illustrated process are highlighted in red and also may show thickened outline when active and broken line when the action is attenuated. Panels A, B, and C presents a simplified schematic of two basic types of CSF infection, *transitory *and *autonomous*, along with a combination of these types in *mixed or amplified *infection. Panels D-G apply these models to the relationships of plasma and CSF HIV (Δplasma:CSF) in four of the settings described in this report, including D. the high Δplasma:CSF in subjects with pleocytosis >10 cells/μL related to exuberant transitory infection; E the high Δplasma:CSF in ADC patients due to enhanced autonomous infection; F. the low Δplasma:CSF in subjects with < 50 blood CD4 cells/μL related to reduced transitory infection; and G the low Δplasma:CSF in treatment failures also related to decreased transitory infection.

To conceptualize CSF changes during infection, we framed 2 simple models of the exchange of HIV and lymphocytes between the blood and CNS compartments [9,14]. Figure 7 provides a schematic diagram of the elements of this model and examples of the variable relationships of CSF to plasma HIV noted in different clinical settings. The model invokes two basic types of infection: *transitory *and *autonomous*, as pictured in Panels A and B of Figure 7.

*Transitory infection *(Figure 7A) refers to infection sustained by short-lived CD4+ T cells trafficking into the CSF space from the blood. In simplified terms, activation of lymphocytes outside of the nervous system favors their promiscuous entry [43,27], and when infected by HIV, these activated CD4 cells can release HIV into the surrounding fluid. This type of infection is determined principally from outside of the CNS, and depends upon systemic infection and cell activation. Infected and uninfected cells are *pushed *into the CSF.

*Autonomous infection *(Figure 7B) refers to infection that is sustained within the CNS by longer-lived cells and does not require continuous repletion from the blood. This is the type of infection that is also assumed to be *'compartmentalized' *[44] and is likely sustained by longer-lived cells of the monocyte lineage that assume residence as perivascular and meningeal macrophages [45] rather than trafficking lymphocytes [46]. Whether such infection is fully self-sustaining or requires renewal from outside remains uncertain [47], but in it, the rates of turnover are lower and the cellular pools are different from those of transitory infection. When accompanied by CSF pleocytosis, the reactive cells are *pulled *into the CSF space [48]. Both of these basic types of infection may occur simultaneously or sequentially, and indeed *mixed infection *with varying contributions of the two types may be the rule (Figure 7C).

We hypothesize that *transitory infection *predominates during the early phases of systemic HIV infection but gives way to an increasing component of *autonomous infection *as immune deregulation progresses. One reason for hypothesizing the early predominance of transitory infection is that the rapid, early-phase decay of CSF HIV RNA noted in some subject initiating therapy is equivalent to that occurring in the plasma. The rate of plasma HIV decay depends upon the potency of the antiviral regimen [49], and as drug exposure in the CNS is almost always lower than at most systemic sites (by virtue of the blood-brain and blood-CSF barriers and other factors reducing drug penetration), we would expect the potency of the regimen in CSF VL to be lower. However, incorporating a model of transitory CSF infection, suppression of systemic infection would also lower the infection rate of trafficking lymphocytes and virus-induced activation, thereby reducing the influx of these cells and rapidly decreasing the CSF VL. Evolution from *transitory *to more *autonomous *infection at later stages of disease would explain the slower CSF decay relative to plasma, as decay kinetics are now closely associated with reduction in T cells. CSF infection may also be *amplified *by entry of activated infected or target CD4 cells which increase their transit across the endothelial barrier in response to chemokines and other signals (Panel C of Figure 7). *Amplified infection *might obscure the underlying importance of autonomous infection as the inciting process, and might respond more readily to systemic treatment.

Variations of these models can also be invoked to explain the relationships of CSF to plasma VL noted in some of the subject groups in this study. For example, the two situations where we found smaller Δplasma:CSF. values might relate to quite different mechanisms. Specifically, in the first instance the association of pleocytosis (>10 cells/μL) with high CSF HIV RNA (approaching that of the plasma VL with both ≥ 10,000 cpm), and small Δplasma:CSF might relate to exuberant cell entry and a high level of transitory or amplified infection as diagrammed in Figure 7D. This mechanism would also account for high CSF HIV concentrations in patients with meningitis due to other infections such as *Mycobacterium tuberculosis *and *Cryptococcus neoformans *[4,50], where cell entry is driven by an unrelated process but result in local amplification of CSF infection. In the second instance, narrow or even reversed (negative) Δplasma:CSF is best explained by a high level of autonomous infection within perivascular or parenchymal macrophages in patients with ADC (Figure 7E).

Figure 7 also illustrates hypothetical explanations for the increased Δplasma:CSF noted in two settings: subjects with CD4 counts < 50 cells/μL (F) and those with treatment failure (G). In both cases, a diminished *transitory *component of infection may be involved, either because of insufficient CD4 target cells or reduced signals and responses to cell activation. In the case of low CD4 cells, the reduced *transitory *component uncovers low-level autonomous CSF infection. Of course, comparison of this setting with ADC in which CD4 cells are often decreased underscores a switch from low-level and seemingly benign autonomous infection to more active and 'malignant' macrophage infection with resultant toxic sequelae. This then raises the question of what causes the change in the character of autonomous infection in this setting – a change in the virus population or an alteration in the host? The increased Δplasma:CSF in treatment failure is supported by observations of diminished cell activation in this setting [51], but is this sufficient to reduce CSF HIV to the extent observed? Might reduced activation and lower CSF HIV levels relate to reduced fitness of the resistant viruses [52] or simply to a quantitative decrement in systemic infection?

These considerations also do not fully preclude the importance of intrathecal drug effects. Simply considering extracellular drug levels in CSF compared to plasma may not be sufficient. Intracellular drug activity of nucleoside RT inhibitors might be enhanced within the cells supporting autonomous CNS infection despite lower extracellular drug concentrations than in systemic sites of infection [53]. Subject 4034, with disproportionate virological failure in CSF compared to blood in the face of a poorly penetrating ART regimen, illustrates the potential importance of drug penetration in the presence of autonomous infection. His high CSF VL was remedied by a regimen with greater penetration.

### Neurological implications

These studies have relevance for two clinical neurological disorders related to CNS HIV infection: *aseptic meningitis *and *ADC*. The first is a component of the central focus of the study, while the second stands as an important background issue.

*Aseptic *or *HIV meningitis *is the clinical diagnosis conferred on HIV-infected patients with CSF pleocytosis without alternative cause, irrespective of clinical symptoms or signs such as headache, photophobia and stiff neck [54-56]. We extend the characterization of this aseptic meningitis, defining its virological profile, clinical context and response to therapy. In the cross-sectional sample, CSF pleocytosis was detected in those with CD4 counts above 50 cells/μL, and was associated with plasma VLs near or above 10,000 cpm.

The frequency of pleocytosis in our subjects and their lack of symptoms confirms earlier observations reported at a time when HIV infection was less well characterized [2,3]. Our subjects were queried and assiduously examined for symptoms or signs of meningitis, and tested for associated abnormality or changes in QNPZ-4. None were found, and we were unable to predict the presence of elevated WBC counts from interviews or examinations. Why is HIV-associated pleocytosis asymptomatic when other infections with similar CSF cell counts are often accompanied by clinical symptoms and signs? Presumably, there are differences in the character of the cells, and more particularly in their secreted products, that determine the absence or presence of symptoms.

Since CSF pleocytosis is frequent and characteristically asymptomatic, how does one approach the HIV-infected patient presenting with headache who has an elevated CSF lymphocyte count [56]? When is pleocytosis incidental and headache due to another cause? When can pleocytosis be attributed to the underlying HIV infection and thus not warrant further diagnostic evaluations? Our experience allows some initial answers to these questions. First, pleocytosis related only to HIV is uncommon in those with blood CD4 counts below 50 cells/μL, and therefore an increased CSF cell count in this setting should be suspect. Second, since ART usually eliminates elevated WBC counts, pleocytosis in a treated patient should be regarded similarly, with the additional consideration of adherence to therapy. Third, since headache is rarely provoked by simple HIV-related pleocytosis, a search for another type of CNS infection or process is warranted in such a case. Finally, in chronic pleocytosis without headache, a trial of ART may confirm HIV as the cause if the cell count resolves. Perhaps the term *aseptic meningitis *in this setting should be reserved for those with symptoms or signs accompanying CSF abnormalities. The larger group of individuals with clinically silent elevated CSF cell counts may simply be designated as having *asymptomatic pleocytosis of HIV infection*.

*ADC *is considered to be caused by brain HIV infection, but mediated by 'indirect' mechanisms involving a variety of pathways initiated and sustained by viral and host gene products [57-61]. Infected and uninfected macrophages and perhaps microglia appear to play a central role in these processes [45]. The current study focused on CSF HIV infection and was not designed to more directly assess the relationship of parenchymal brain HIV infection to ADC. Nonetheless, it afforded a view of certain aspects of this important issue.

Neurological performance was assessed using the QNPZ-4 score. This correlated highly with the presence and stage of ADC; the reduced QNPZ-4 score (median -0.50) of the cross-sectional sample was largely the result of including the ADC subjects. Longitudinally, the QNPZ-4 score afforded a stable measure in those without change in clinical neurological status, while it tracked a pattern of improvement in ADC subjects responding to ART that mirrored their antiviral response in CSF and plasma (Figure 6). Cross-sectionally, both ADC stages and QNPZ-4 scores correlated with blood CD4 counts, though neither correlated overall with plasma or CSF HIV RNA levels. The association of ADC with HIV progression and depressed CD4 blood counts has been well documented [62], and the lack of correlation of neurological disease with the magnitude of systemic or CSF infection over the broad range of CD4 counts has also been previously noted [6,7]. Our findings agree with some [8] but differ from other reports [7] in that we also did not find a strong correlation between the neurological and virological measurements in subjects with lower CD4 counts. There was only a weak correlation between CSF VL and ADC stage (p = 0.037, rho = 0.316) in subjects with CD4 counts below 200 cells/μL, and no significant correlation with QNPZ-4 score in this group; no correlation of the CSF HIV concentration with ADC stage or QNPZ-4 score was found in subjects with CD4 counts <50 cells/μL. Moreover, we found no correlation between the neurological measures and CSF VLs among the entire group of untreated subjects, or in those with CD4 counts below 200 cells/μL.

This does not mean that HIV brain infection is not central to the pathogenesis of ADC or that HIV brain infection cannot be reflected in CSF, but only that such an association is obscured by other factors. In this study, there may have been two principal reasons why no association was found. First, there was a high 'background' of increased CSF HIV in neurologically normal subjects. Second, many of our ADC subjects were on ART and clinically stable, and therefore likely suffered 'inactive' or residual brain disease. The lack of diagnostic specificity of CSF HIV RNA measurements underscores the varied relationship between CSF changes, on the one hand, and brain infection and disease, on the other. The CSF space and brain parenchyma are best viewed as two separate but intersecting compartments in which infection is not always congruent, necessitating caution in interpreting brain events from CSF findings. At least three types of intersections may occur. First, brain infection can 'overflow' into the CSF so that brain-derived HIV (and other markers) can be detected and directly measured in this fluid. The likely major cells of origin for HIV in these cases of autonomous infection are perivascular macrophages and perhaps the parenchymal macrophages and microglia [45], with virus reaching the CSF by diffusion along the perivascular spaces. CSF in this setting can provide a direct sample of brain infection. Second, CSF and brain infection may be parallel, although not necessarily identical. Because the leptomeninges and brain are both non-lymphatic organs and separated from the blood by barriers to the free passage of viruses, immune defenses (both humoral and cellular) and drugs, infection and host responses may be sufficiently similar in the meninges and brain to permit CSF analysis to monitor brain infection, whether autonomous or transitory. Third, infections might markedly diverge in these compartments, in which case CSF would provide little direct insight into brain infection; at the extreme, the CSF might simply reflect meningeal infection which has no counterpart within the brain. The problem for the clinician is to distinguish which of these three relationships predominates in a given patient.

Measuring the VL in CSF is not enough to make these important distinctions, and the CSF HIV RNA concentration cannot, in isolation, serve as a reliable diagnostic marker for ADC. Nor can the presence of pleocytosis be used to rule in or out ADC. This is unfortunate since objective markers are needed both in the clinic for practical diagnosis and in the research setting to more precisely define therapeutic targets. In the occasional patient, such as those designated as outliers in Figure 2, substantially higher HIV RNA in CSF than in plasma may be diagnostically suggestive, but in most cases neither the level of CSF virus nor its relation to the plasma VL distinguishes those with ADC. Future efforts need to assess the value of supplementary CSF measurements, involving more detailed characterization of the virus (cell tropism, chemokine co-receptor utilization, or still-elusive markers of neuropathogenicity) [63-65] along with the use of ancillary markers of immune responses and neural injury [8,66,67].

An additional clinical need is for laboratory measures that assess the *activity *of ADC and underlying HIV encephalitis. While we included subjects in this study with treated ADC and suppressed CSF infection, this would not be appropriate in a clinical trial assessing ADC treatment. Measuring CSF HIV concentration may be helpful in this setting, since undetectable CSF HIV likely signals suppressed brain infection.

## Conclusion

HIV infection of the CNS is a nearly ubiquitous facet of systemic infection, but varies in character and clinical consequences. From very early exposure during primary systemic infection, most HIV-infected patients experience chronic asymptomatic CNS infection. However, a few individuals will develop encephalitis presenting as ADC. CSF sampling provides a valuable window into this infection and its variability.

We have framed the discussion of our results in models of CSF infection. Embedded in these models are a number of dichotomies relating systemic to CNS infections: (1) *transitory *versus more *autonomous *infections with rapid versus slower turnover rates; (2) CSF lymphocytosis either *causing *or *responding to *local infection, and (3) infection of the meninges presenting as *'asymptomatic pleocytosis' *versus more toxic parenchymal or perivascular infection leading to the brain dysfunction of ADC. These provide a framework for future studies examining the mechanisms of infection in molecular terms and with respect to cell and HIV exchange and compartmentalization.

On a more practical level, to the extent that CSF infection reflects infection of the brain, antiretroviral therapy is usually effective in suppressing CNS HIV replication. Our longitudinal observations show that CSF infection usually responds well to combination antiretroviral therapy, equaling or exceeding systemic responses as reflected in plasma. Even where resistance leads to virological failure and persistent plasma viremia, ART may have a salutary effect on CSF. While the mechanisms underlying these favorable treatment effects remain uncertain, these observations are consonant with other reports using less frequent monitoring and are therapeutically reassuring. Our findings suggest that favorable virological outcomes in the CSF are the rule rather than an exception. They are also consistent with clinical studies that report a falling incidence of ADC in the current treatment era [68].

## Competing interests

The author(s) declare that they have no competing interests.

## Authors' contributions

SS participated in analysis and interpretation of the data and helped to draft the manuscript. AN helped with subject evaluations. NL participated in analysis and preparation of the data. TL helped in the study planning and coordinated the viral load assays. CP participated in planning of the genotypic and phenotypic resistance studies. SD participated in the study planning and design. EP participated in planning and interpretation of the genotypic and phenotypic resistance studies, and helped prepare the manuscript. RWP conceived of the design and analysis of the study and drafted the manuscript.

## Pre-publication history

The pre-publication history for this paper can be accessed here:



## Supplementary Material

Additional File 1 Table 2. Clinical data, ART regimen history, and genotypic analysis of resistance mutations from five subjects with dissociated treatment responses. Antiretroviral medications able to penetrate into the CSF [10] are indicated in bold font. Mutations are given as differences from a drug-sensitive control (e.g., NL 4–3). Wild-type amino acids are indicated by the single capitalized letters in black while substitutions are indicated by either red or blue font. In red, but not bold, are minor mutations associated with resistance to a drug the subject was taking at the time of sampling. However, red and bolded type indicates major mutations associated with resistance to a drug that the subject was taking at the time of sampling. In blue, not bold, are minor mutations associated with resistance to a drug that the subject was not currently taking, while blue-bold typeface indicates major mutations associated with resistance to a drug which subject was not taking at the time. Click here for file
